# Radial nerve palsy in humeral shaft fractures with internal fixation: analysis of management and outcome

**DOI:** 10.1007/s00068-017-0775-9

**Published:** 2017-03-09

**Authors:** T. R. Schwab, P. F. Stillhard, S. Schibli, M. Furrer, C. Sommer

**Affiliations:** Kantonsspital Graubünden, Loestrasse 170, 7000 Chur, Switzerland

**Keywords:** Humeral shaft fracture, Primary radial nerve palsy, Secondary radial nerve palsy, Internal fixation

## Abstract

**Introduction:**

The incidence of radial nerve injury after humeral shaft fractures is on average 11.8% (Shao et al., J Bone Jt Surg Br 87(12):1647–1652, 2005) representing the most common peripheral nerve injury associated with long bone fractures (Korompilias et al., Injury, 2013). The purpose of this study was to analyze our current policy and long-term outcome, regarding surgically treated humeral shaft fractures in combination with radial nerve palsy.

**Materials and methods:**

We retrospectively analyzed the data of patients with surgically treated humeral shaft fractures from 01/01/2003 to 28/02/2013. The analysis included fracture type, soft tissue injury regarding closed and open fractures, type of fixation, management, and outcome of radial nerve palsy.

**Results:**

A total of 151 humeral shaft fractures were fixed in our hospital. In 20 (13%) cases, primary radial palsy was observed. Primary nerve exploration was performed in nine cases. Out of the 13 patients with follow-up, 10 showed a complete, 2 a partial, and 1 a minimal nerve recovery. Two of them underwent a revision procedure. Secondary radial nerve palsy occurred in 9 (6%) patients postoperatively. In five patients, the radial nerve was not exposed during the initial surgery and, therefore, underwent revision with nerve exploration. In all 5, a potential cause for the palsy was found and corrected as far as possible with full recovery in 3 and minimal recovery in one patient. In four patients with exposure of the nerve during the initial surgery, no revision was performed. All of these 4 showed a full recovery.

**Conclusion:**

Our study showed an overall rate of 19% radial nerve palsy in surgically treated humeral shaft fractures. Most of the primary palsies (13%) recovered spontaneously, and therefore, nerve exploration was only exceptionally needed. The incidence of secondary palsy after surgery (6%) was high and mainly seen after plate fixation. In these cases, we recommend early nerve exploration, to detect and treat potential curable neural lesions.

## Introduction

One of the most common reasons for peripheral nerve palsy is an injury of the radial nerve associated with a fracture of the humeral shaft [1–4]. Regarding the literature, the overall incidence of radial nerve palsy in patients with humeral shaft fracture is between 7 and 17% [3]. One can differ between primary or traumatic nerve injury and secondary or iatrogenic nerve injury following fracture fixation or manipulation [3, 5, 6]. Claessen et al. found an incidence of 7% for iatrogenic radial nerve palsy in patients with humeral shaft fractures, and data in the literature vary between 6 and 32% [6–8].

Regarding treatment, no clear consensus exists regarding if and when the nerve should be explored surgically. Earlier studies have reported a high rate of spontaneous recovery in patients with primary nerve injury: A “wait and see” strategy seems to be widely accepted, and early nerve exploration is only recommended in special situations (e.g., open fractures) [1, 6, 9–11]. In contrast, the opinions differ about necessity of early nerve exploration in patients suffering from secondary nerve palsy after the initial surgical fracture fixation. While some authors recommend early exploration [12], others advocate for a 4 or 6 month observation period [3, 5]. According to literature, there is no significant difference in overall recovery rate in primary radial nerve palsy (88%) and secondary radial nerve palsy (93%) [1].

Despite the lack of clear evidence for early exploration of secondary radial nerve palsy [6], we pursue this policy at our clinic. The purpose of this study was to retrospectively analyze treatment and long-term outcome of primary and secondary radial nerve palsy, observed in surgically treated humeral shaft fractures in a Swiss Level A Trauma Center within a 10-year period. In particular, we wanted to investigate whether a policy with early exploration for secondary radial nerve palsy, within a maximum of 2 weeks after trauma, is justified. In all patients with secondary palsy, which underwent early exploration, we found a potential cause for the palsy which we were able to treat surgically. We suppose that early exploration in secondary nerve palsy offers a chance for an earlier start of nerve rehabilitation and, therefore, leads to better outcomes.

## Materials and methods

After the ethic committee approval (Kantonale Ethikkommission Zürich, 8090 Zurich, Switzerland, SNCTP000000337), all patients with surgically treated humeral shaft fractures treated at our institution during a 10-year period (01/01/2003–28/02/2013) were included. Patient charts, operation, and follow-up notes were retrospectively analyzed. Patients with pathologic humeral shaft fractures were excluded. Fracture fixation was done with plate osteosynthesis (POS) or intramedullary nailing (IM). The variables collected are shown in Tables 1, 2, 3, and 4. The data were transferred into an anonymized chart using a standard spreadsheet software (Microsoft Excel).

**Table 1:** “Primary radial nerve palsy” patients’ characteristics and pattern of injury

Patients with primary radial nerve palsy (*n* = 20)			
Age (years)	40 (17)	Fracture side left/right (*n*)	10/10
Sex: male/female (*n*)	11/9	AO 12–A/B/C (*n*)	7/8/5
BMI (kg m^−2^)	24 (5)	Open fractures (*n*)	5
ASA 1/2/3 (*n*)	8/10/2	High-energy trauma (*n*)	8

Data are expressed as mean (standard deviation) and numbers

*AO* fracture classification by “Arbeitsgemeinschaft für Osteosynthesefragen”, *ASA* American Society of Anesthesiologists classification of physical status, *BMI* body mass index

**Table 2:** “Secondary radial nerve palsy” patients’ characteristics and pattern of injury

Patients with secondary radial nerve palsy (*n* = 9)			
Age (years)	45 (20)	Fracture side left/right (*n*)	4/5
Sex: male/female (*n*)	100/0	AO 12–A/B/C (*n*)	4/2/3
BMI (kg m^− 2^)	25 (6)	Open fractures (*n*)	1
ASA 1/2/3 (*n*)	3/4/2	High-energy trauma (*n*)	4

Data are expressed as mean (standard deviation) and numbers

*AO* fracture classification by “Arbeitsgemeinschaft für Osteosynthesefragen”, *ASA* American Society of Anesthesiologists classification of physical status, *BMI* body mass index

**Table 3:** Treatment and outcome group “primary radial nerve palsy”

	Implant	Surgical approach	Exploration of radial nerve	Macroscopic nerve lesion	Revision surgery	Grade of nerve injury	Clinical course	Follow-up (months)
p1	LCP	Open dorsal	No		No	Motor + sensory	Complete recovery	32
p2	LCP	Open dorsal	Primary	Contusion	No	Motor + sensory	–	Lost
p3	IM	Percutaneous, retrograde (initial ex fix)	No		No	Motor + sensory	Complete recovery	17
p4	IM	Percutaneous retrograde (initial ex fix)	No		No	Motor + sensory	–	Lost
p5	IM	Percutaneous, retrograde	No		No	Sensory	Complete recovery after 1.5 months	14
p6	IM	Percutaneous, retrograde	No		No	Motor + sensory	Complete recovery after 3 months	3
p7	LCP	Open dorsal	Primary	Contusion	Decompression	Motor + sensory	Complete recovery after 3 months	3
p8	IM	Percutaneous, retrograde	No		No	Motor + sensory	Partial recovery	6
p9	LCP	Open dorsal	Primary	No	No	Motor + sensory	–	Lost
p10	LCP	Open dorsal	Primary	Contusion	No	Motor + sensory	–	Lost
p11	IM	Percutaneous, retrograde	No		No	Motor + sensory	–	Lost
p12	LCP	Open deltoido-pectoral	No	–	No	Motor + sensory	Complete recovery	20
p13	LCP	Open dorsal (initial ex fix)	Primary	90% intact, 10% ruptured	No	Motor + sensory	Complete recovery after 16 months	31
p14	LCP	Open deltoido-pectoral	Primary	No	No	Motor + sensory	–	Lost
p15	LCP	Open dorsal	Primary	No	No	Motor + sensory	Complete recovery after 8 months	8
p16	IM	Percutaneous retrograde	Secondary	Traumatic cut	Yes, after 7 months nerve graft tendon transfer after 16 months	Motor + sensory	Initial progression of Tinel’s response, then stop	16
p17	IM	Dorsal distal	No		No	Motor + sensory	Complete recovery after 6 months	20
p18	LCP	Open dorsal	Primary	No	No	Motor + sensory	Complete recovery after 1 months	14
p19	IM	Percutaneous retrograde	No		No	Motor + sensory	–	Lost
p20	LCP	Open dorsal	Primary	No	No	Motor + sensory	Partial recovery	13

*IM* intramedullary nailing, *LCP* locking compression plate, *MIPO* minimal invasive plate osteosynthesis

**Table 4:** Treatment and outcome group “secondary radial nerve palsy”

	Implant	Initial surgical approach (1.Op)	Exploration of radial nerve	Macroscopic nerve lesion	Revision surgery	Grade of nerve injury	Clinical course	Follow-up (months)
s1	LCP	MIPO, delta-split, open distally (no primary nerve exploration)	Secondary	Contusion, scratched, irritation by plate	Day 6 postop.: decompression	Motor + sensory	Complete recovery after 11 months	14
s2	Ex fix	Percutaneous	Secondary (during change from ex fix to plate)	10% lacerated by ex.-fix. pin		Motor + sensory	Complete recovery after 12 months	12
s3	LCP	Open, dorsal	Primary	No		Motor + sensory	Complete recovery (unclear when)	41
s4	LCP	MIPO, delta-split, distally open	Primary	No		Motor + sensory	complete recovery after 12 months	12
s5	LCP	MIPO, delta-split, distally open	Primary	nerve trapped under plate, immediate correction, contusion		Motor + sensory	–	Lost
s6	IM	Percutaneous antegrade	Secondary	Complete transection by distal locking bolt	Day 6 postop.: exploration, day 13 postop.: 70 mm nerve graft	Motor + sensory	No recovery after 1.5 year → tendon transfer	18
s7	LCP	Open distally, delta-split proximal	Secondary	Nerve under plate, contusion	Day 5 postop: Revision	Motor + sensory	Partial recovery, decreased strength and persisting sensatory deficit	24
s8	LCP	Open distal dorsal, local nerve exposure prox. the fracture	Primary	No		Sensory	Complete recovery after 7 months	7
s9	LCP	Open, dorsal	Secondary	Contusion by bone fragment	Day 9 postop.: new plate fixation, removal of bone fragment	Sensory	Complete recovery	3

*IM* intramedullary nailing, *LCP* locking compression plate, *MIPO* minimal invasive plate osteosynthesis

Any documented sensory radial nerve palsy or a weakness of the wrist extensors or drop hand described in the medical records on admission or during hospitalization was defined as radial nerve palsy. For the fracture type, the AO classification was used, for grading of the soft tissue damage, the Anderson/Gustilo classification for open fractures and the Tscherne/Oestern classification for closed fractures were used [13–16]. Regarding trauma mechanism, we differentiated between high-energy and lower energy trauma. As high-energy trauma, we regarded high velocity car, motorbike, ski, snowboard, and paragliding accidents, as well as ≥2° open fractures or ≥G2 soft tissue damage.

We analyzed patients in two independent groups: patients with primary or traumatic radial palsy presenting loss of radial nerve function at admission (group 1), and patients with secondary or iatrogenic radial palsy, presenting loss of radial nerve function after surgical fracture treatment (group 2). The documented recovery of radial nerve function during follow-up was assessed clinically including objective data such as electromyography and neurography (EMG/ENG) if present.

For patients’ characteristics, the mean value with standard deviation was calculated, the follow-up is described as median with range. For statistical evaluation, the recovery rate was calculated as percentage.

## Results

During the observed period, a total of 151 patients with a fracture of the humeral diaphysis underwent surgery with internal fracture fixation as a definitive treatment. 59% received a plate osteosynthesis (POS) and 41% of the fractures were fixed by intramedullary nailing (IM). Regarding AO classification [16], among patients treated with POS, 44% fractures were classified as type C, 29% as type B and 27% as type A. In patients treated with IM, 8% were graded as type C, 29% as type B, and 63% as type A (Fig. 3). Patients’ mean age was 49 (21) years.

13% (20 out of 151) of all patients showed primary radial nerve palsy. Table 1 shows patients’ characteristics in this group. In 45% (9 out of 20), all of them patients with POS, an early exploration of the radial nerve was performed during the initial surgery. In one patient with a closed transverse fracture after high-energy direct trauma (IM), a secondary nerve exploration was performed during revision surgery 7-month post-trauma and a traumatic cut of the radial nerve was found at the fracture level. In 50% (10 out of 20), there was no nerve exploration at all (Fig. 4; Table 3).

In 44% (4 out of 9) with primary radial nerve palsy and the initial exploration, minor nerve injuries such as contusions or superficial damage were found. In one patient, the surgeon decided to perform decompression, because the radial nerve seemed to be entrapped by tight soft tissue structures (intermuscular septum) in addition to the damage by the fracture itself. In 56% (5 out of 9), no macroscopic injury was found.

Median follow-up in this group was 14 (3–32) months. 35% (7 out of 20) were lost during the follow-up due to foreign domicile. Within the group of patients with primary palsy and follow-up, 77% (10 out of 13) showed a complete and 15% (2 out of 13) partial recovery of radial nerve function without further surgical interventions (Fig. 5). The one patient suffering from a traumatic cut underwent several revision procedures. Unfortunately, an initial progression of the Tinel’s response from the fracture localization 20 cm above the elbow joint till 8 cm above elbow was misinterpreted as nerve recovery. As there was no further progression of the Tinel’s response and no functional nerve recovery, 7-month post-trauma, the revision was performed and a nerve graft was implanted. Finally, without satisfying recovery of the nerve function after more than 1 year, a tendon transfer operation was carried out.

6% (9 out of 151) of all patients suffered from secondary radial nerve palsy. Table 2 shows patients’ characteristics in this group. In 78% (7 out of 9), the radial nerve palsy occurred after POS, in 11% (1 out of 9) after IM and in 11% (1 out of 9) after temporary fracture fixation with an external fixator. In 44% (4 out of 9) of these patients with secondary nerve palsy, radial nerve exposure was performed at the time of the initial surgery due to the chosen approach for fracture fixation (2× dorsal ORIF,^1^ 2× anterolateral MIPO^2^). In all these cases, an intact nerve was initially observed, and therefore, no further revision procedure was performed despite the fact that secondary palsy occurred. In the remaining 56% (5 out of 9), in which the radial nerve was not exposed during the initial surgery, a revision surgery was performed within a maximum of 9 days after the initial intervention (Fig. 6; Table 4).

**Fig. 1:**
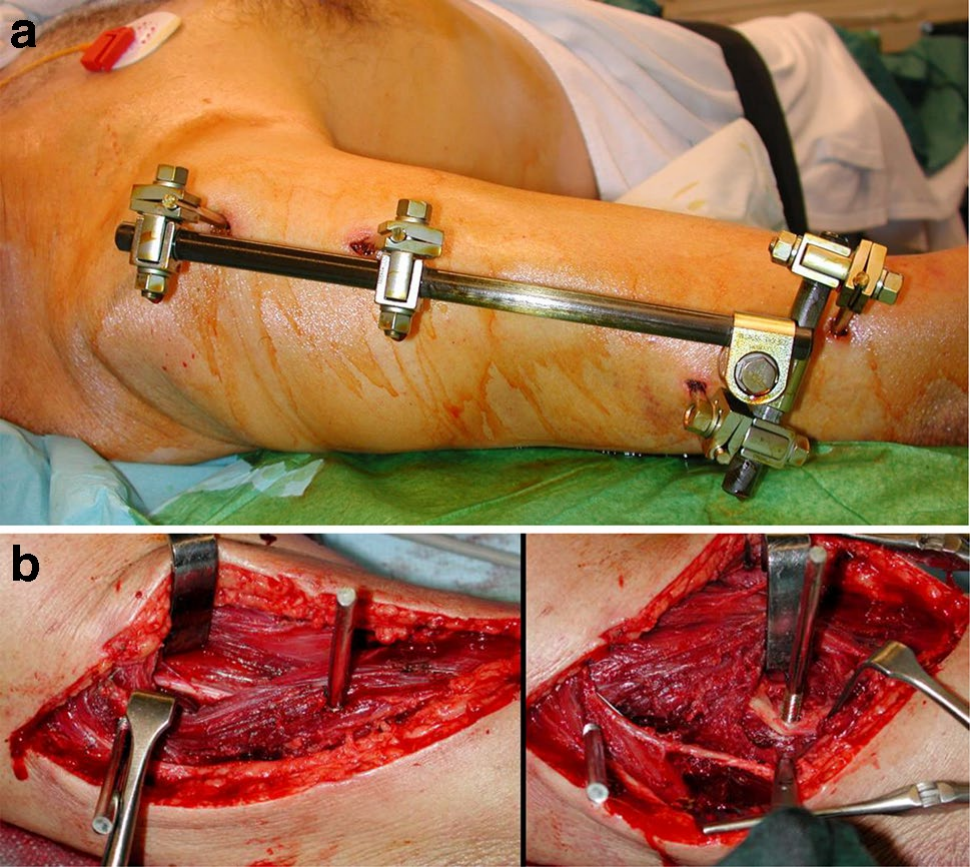
**a** 62 years, car accident, secondary nerve palsy after application of an external fixator, **b** 62 years, distal pin runs through brachioradial muscle winding up the radial nerve and destroyed 10% of its circumference

**Fig. 2:**
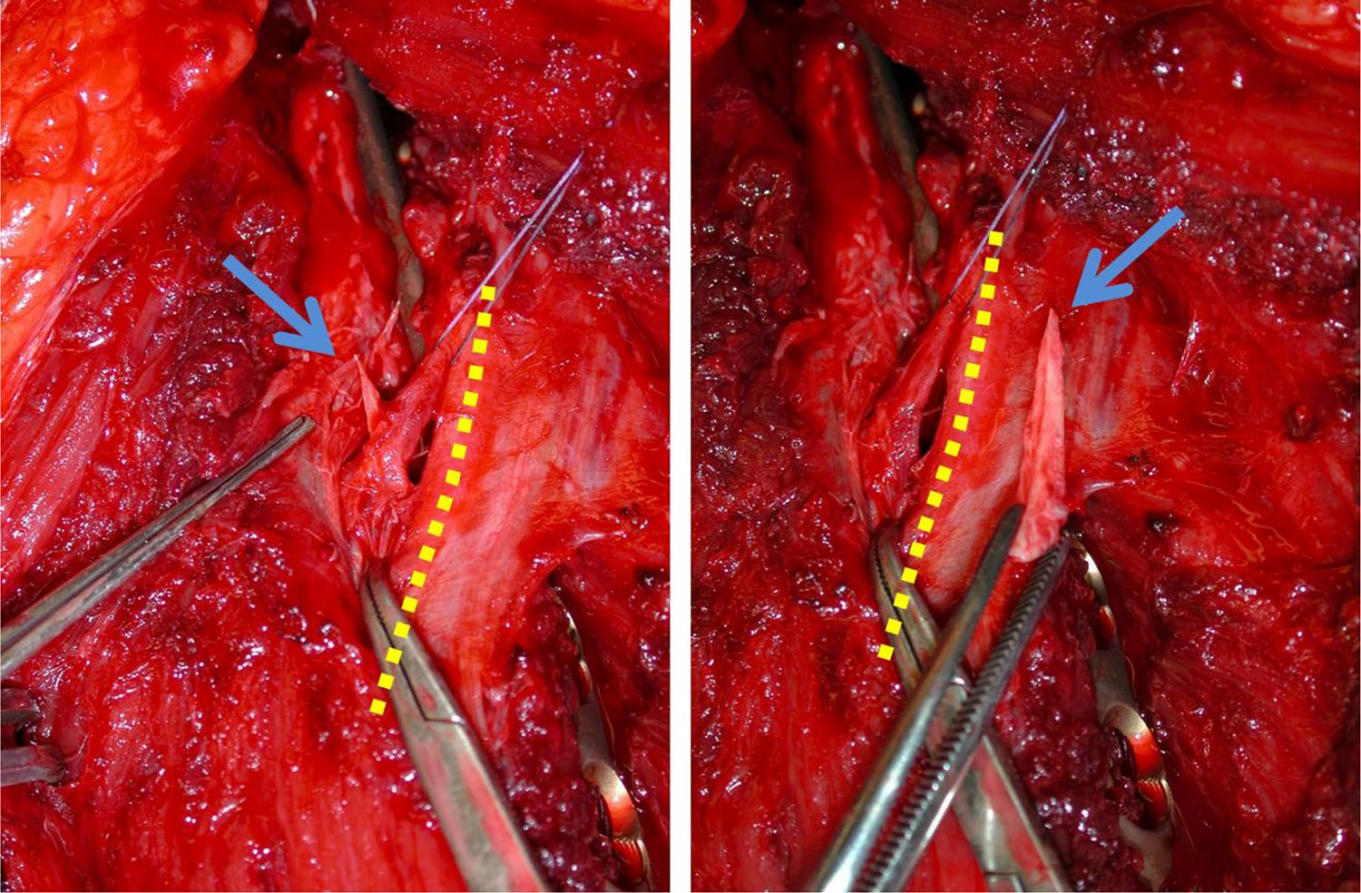
19 years, secondary nerve palsy after POS; bony fragment (*blue arrow*) sticking in a perineural small artery, adjacent to the radial nerve (*yellow dotted line*)

In all patients who underwent surgical revision, macroscopic nerve damage was found: damage by a pin of the external fixator (Fig. 1a, b), compression by a bone fragment (Fig. 2), entrapment between plate and bone, irritation or displacement by the plate, and damage by a distal locking bolt of the intramedullary nail were among the causes of these nerve injuries.

**Fig. 3:**
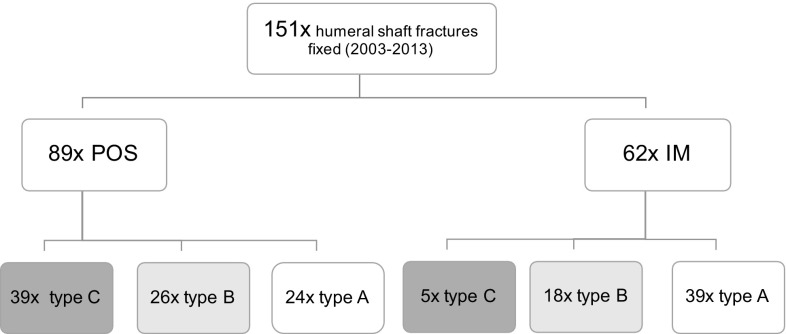
Distribution of surgically treated fractures regarding AO fracture classification [16]. Data are expressed as numbers and fracture type (AO 12-x); *AO* fracture classification by “Arbeitsgemeinschaft für Osteosynthesefragen”, *IM* intramedullary nailing, *POS* plate osteosynthesis

**Fig. 4:**
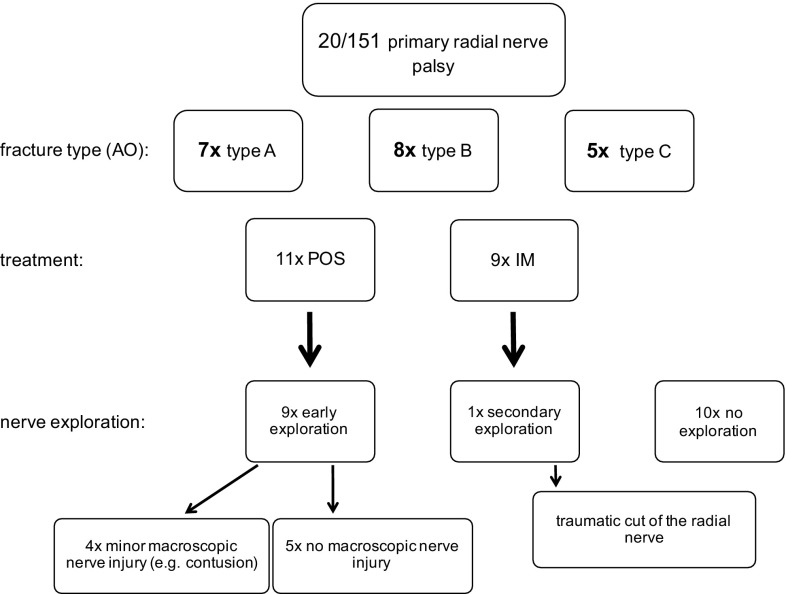
Treatment and nerve exploration in primary radial nerve palsy patients. Data are expressed as numbers; *AO* fracture classification by “Arbeitsgemeinschaft für Osteosynthesefragen”, *IM* intramedullary nailing, *POS* plate osteosynthesis

**Fig. 5:**
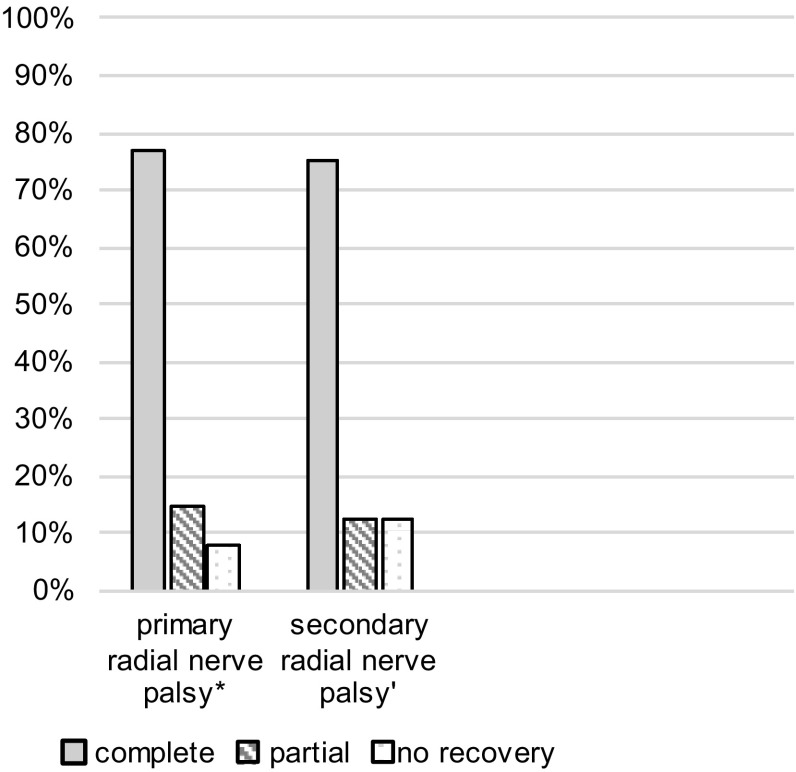
Recovery rate of nerve function in primary and secondary radial nerve palsy. Data are expressed as percentage: (*asterisk*) *n* = 13, median follow-up 14 (3–32) months (7 patients lost to follow-up); (*single quote*) *n* = 8, median follow-up 13 (3–41) months (1 patient lost to follow-up)

**Fig. 6:**
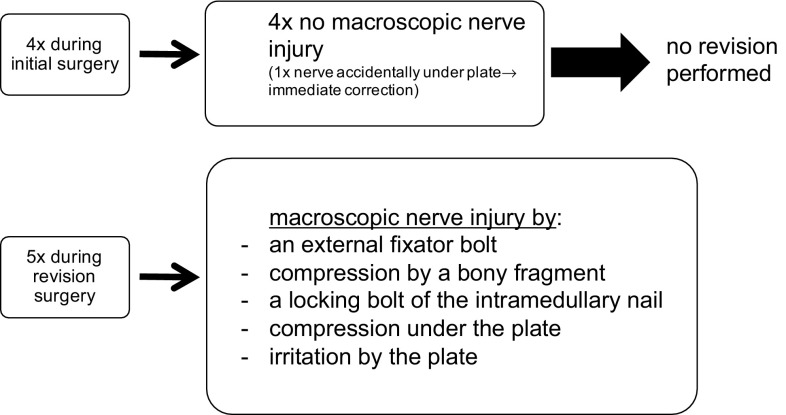
Radial nerve exploration and findings in secondary radial nerve palsy

**Fig. 7:**
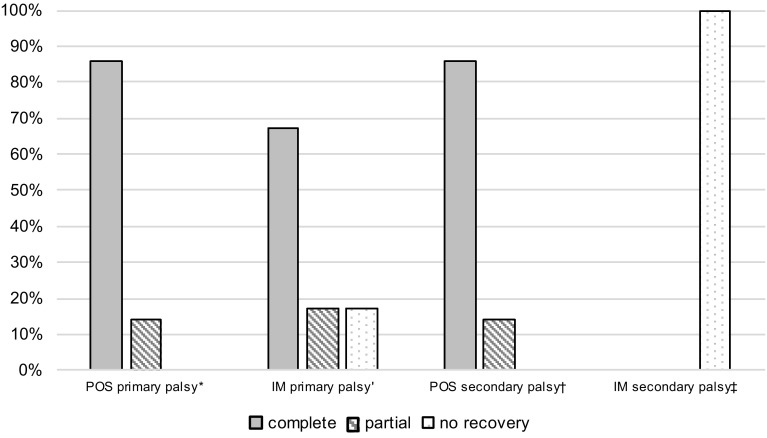
Recovery rate of nerve function in consideration of the fracture treatment. Data are expressed as percentage: (*asterisk*) *n* = 7 (7 patients lost to follow-*up*); *n* = 6; (*dagger*) *n* = 7 (1 patient lost to follow-*up*); (*double dagger*) *n* = 1; *IM* intramedullary nailing, *POS* plate osteosynthesis

**Fig. 8:**
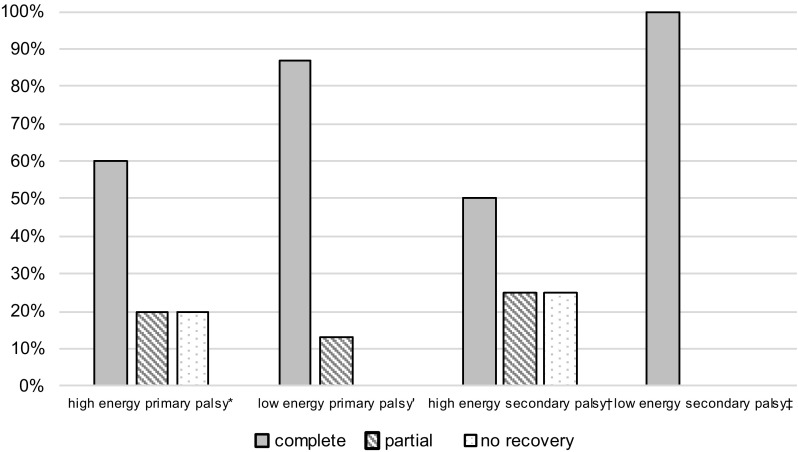
Recovery rate of nerve function in consideration of the trauma energy. Data are expressed as percentage: (*asterisk*) *n* = 5 (3 patients lost to follow-*up*); *n* = 8 (4 patients lost to follow-up); (*dagger*) *n* = 4; (*double dagger*) *n* = 4 (1 patient lost to follow-up)

During revision procedures, the probable cause for the secondary palsy could be corrected: the bone fragment was removed (Fig. 2), the compressed nerves were released, and the plates repositioned and the transected nerve fixed by an early neural graft.

Median follow-up in this group was 13 (3–41) months. 11% (1 out of 9) were lost to follow-up. 75% (6 out of 8) showed a complete recovery of nerve function (Fig. 5). The one patient with the nerve compressed under the plate only showed partial recovery of nerve function, despite releasing the nerve within only 5 days after the initial surgery. The one patient with palsy after IM presented a major nerve damage of approximately 7 cm, probably due to winding up the nerve with the locking bolt. Despite nerve graft implantation and several revision procedures, a tendon transfer had to be performed ultimately.

Regarding nerve recovery depending on fixation technique, primary palsy treated with POS showed an 86% (6 out of 7) complete recovery rate. Primary palsy treated with IM showed a 67% (4 out of 6) complete recovery rate. Secondary palsy definitively treated with POS showed complete recovery in 86% (6 out of 7) of the cases and the one patient with IM did not show any recovery. Data are visualized in Fig. 7. Thirty-five percent (7 out of 20) were lost to follow-up.

Regarding trauma energy in patients with primary radial nerve palsy, 40% (8 out of 20) suffered from high-energy trauma. Out of these, 60% (3 out of 5) showed complete recovery. Without high-energy trauma, 88% (7 out of 8) recovered completely (Fig. 8). Thirty-five percent (7 out of 20) were lost to follow-up.

Regarding patients with secondary nerve palsy, 44% (4 out of 9) suffered from high-energy trauma, from which 50% (2 out of 4) recovered completely. Without high-energy trauma, 100% (4 out of 4) of patients showed a complete recovery (Fig. 8). Eleven percent (1 out of 9) were lost to follow-up.

## Discussion

Regarding primary or traumatic radial nerve palsy associated with humeral shaft fractures, our study showed a high rate of spontaneous complete recovery of nerve function (77%), correlating well with the literature [1, 10, 11, 17]. Early exploration of the radial nerve in primary traumatic palsy does not seem to be necessary, especially in closed fractures, where a primary serious nerve damage requiring surgical repair is very rare. In our collective, this was true for only one patient (see below). Out of nine patients who underwent early exploration, only in one case, a decompression of the nerve, at the level where it passes the intermuscular septum, seemed to be indicated and was performed. The other cases with early exploration only showed minor to no damage and no possibility of surgical treatment. Out of the eight patients with follow-up after primary palsy without nerve exploration at the time of surgery, six showed a complete and one patient a partial recovery. Only in one case after high-energy direct trauma with a transverse fracture, a complete transection of the nerve at fracture level was detected 7-month post-trauma during revision surgery while failing to regain nerve function. Despite nerve grafting, major motoric deficits remained requiring a tendon transfer later on.

The low risk of primary neural damage requiring surgical repair (8%) does not justify an early nerve exploration in all the cases with nerve palsy. It could be a viable option in certain cases such as open fractures or cases with high-energy trauma [18, 19]. Furthermore, a primary nerve palsy should not influence the decision between conservative or operative treatment and the choice of implant being either a plate or a nail [3].

Several limitations regarding our conclusions have to be pointed out: with our small case load and the large number of patients lost to follow-up (35%), further statistical analysis with scientific validity is not reasonable, but our observations equate conclusions drawn from earlier studies [1].

Claessen et al. found radial nerve palsy in humeral shaft fractures significantly associated with high-energy trauma, open fractures, as well as surgical approach [8]. Considering the low number of patients in these subgroups, differences in outcome comparing osteosynthesis methods and trauma energy in our study (Figs. 7, 8) have to be interpreted with caution and no definitive conclusions can be drawn from these observations.

Dealing with secondary radial nerve palsy detected after an surgical fracture fixation, we believe that an exploration of the radial nerve, if not already done during the first surgery, is obligatory and should be performed shortly after the first operation: With a 6% rate (9 patients out of 151) of postoperative or iatrogenic radial nerve injury, we had a slightly lower complication rate than average [4, 6, 8]. The impairment in everyday life as well as the psychological strain for patients suffering from a radial nerve palsy are severe and should be avoided if possible [18, 20]. This implies an explicit patient information preoperatively.

In four patients with secondary palsy, the radial nerve was initially explored during definitive fracture fixation due to the surgical approach (posterior or anterolateral distally, Table 4). As to be expected, the nerve was found to be intact. In these four cases, no early revision was performed, and in the three patients with follow-up, a full recovery was observed. There are several risks and reasons for a secondary radial palsy during fracture fixation: even light tension to the nerve during exploration might cause neurapraxia. Another potential source for injury is the positioning of the patient before and during surgery. Under general anesthesia, the loss of consciousness along with muscle tone might lead to accidental and excessive movement at the fracture site. Similar traction damage can occur because of excessive indirect fracture manipulation during nailing. A strong tension to the surrounding radial nerve or an entrapment between bone fragments can be the consequence [4–6, 21]. Therefore, a temporary fracture immobilization, e.g., with a cast and attention during anesthetic induction and patient positioning, of both the surgeon and the support personal have to be guaranteed. Great caution should be taken during the approach and the fracture reduction [8]. In the critical region especially from the middle to the distal humeral shaft, where the radial nerve transverses the intermuscular septum, special retraction devices such as Hohmann retractors are dangerous. Their use should be avoided.

In all the five patients with secondary radial palsy after fracture fixation without exploration of the radial nerve, we went for early revision with exploration of the radial nerve (Fig. 6; Table 4). In all these patients, macroscopic nerve damage was found and an appropriate treatment was applied: In one case, a sharp spiky bone fragment traversing the radial nerve was removed (Fig. 2). In three cases after partially percutaneous anterolateral plate fixation, the entrapped or compressed nerves were released and two plates had to be newly positioned. In one case, after antegrade nail fixation, a nerve was found to be transected at the level of the distal locking bolts, most likely caused by the percutaneous drilling maneuver. This nerve was repaired by an early neural graft, unfortunately without great success. Because of this experience, we believe that our approach with early nerve exploration for secondary radial nerve palsy is reasonable. Due to scar tissue, a revision surgery with a maximum of 10–14 days after the initial surgery seems to be more easy than 3–4 months later as it is proposed by other authors [4, 6, 22]. With a total recovery rate of 75% in secondary nerve palsy, we observed a similar rate comparing it to primary radial nerve palsy (77%). It remains questionable whether the recovery rate would have been the same without early exploration in the five patients mentioned above. It can be assumed that rehabilitation would have been postponed and a later on secondary exploration would have been necessary.

Since all patients with secondary nerve palsy underwent nerve exploration within 9 days after trauma, a statement about the outcome without early exploration cannot be made.

In conclusion, we think that a “wait and observe” strategy for a potentially compressed or damaged radial nerve is wrong. We recommend early nerve exploration in patients with secondary radial nerve palsy in which the nerve had not been exposed during the initial surgery.

## Footnotes

1 ORIF: open reduction, internal fixation.

2 MIPO: minimal invasive plate osteosynthesis.
